# Supplementary material for: Octenidine Lozenges Intended for Oral Administration Display In Vitro Activity Against Oropharyngeal Pathogens and Safety Toward Intestinal Microbiota
Source: Int J Mol Sci. 2025 Oct 15;26(20):10045. doi: 10.3390/ijms262010045 (PMC12563938; doi:10.3390/ijms262010045)
Supplement: Supplementary file 1 [file ijms-26-10045-s001.zip › ijms-3869725-supplementary.pdf]

	Results compliance [Y/N]	
	OCT-lozenge	Pure OCT
S. aureus 29213	Y	Y
P. aeruginosa 27853	Y	Y
C. albicans 10231	Y	Y

Table S1. Comparison of compliance of antimicrobial results of the same concentrations of octenidine from lozenges vs powder. Micro-plate method for MIC assessment; 24 hour contact/exposure time.

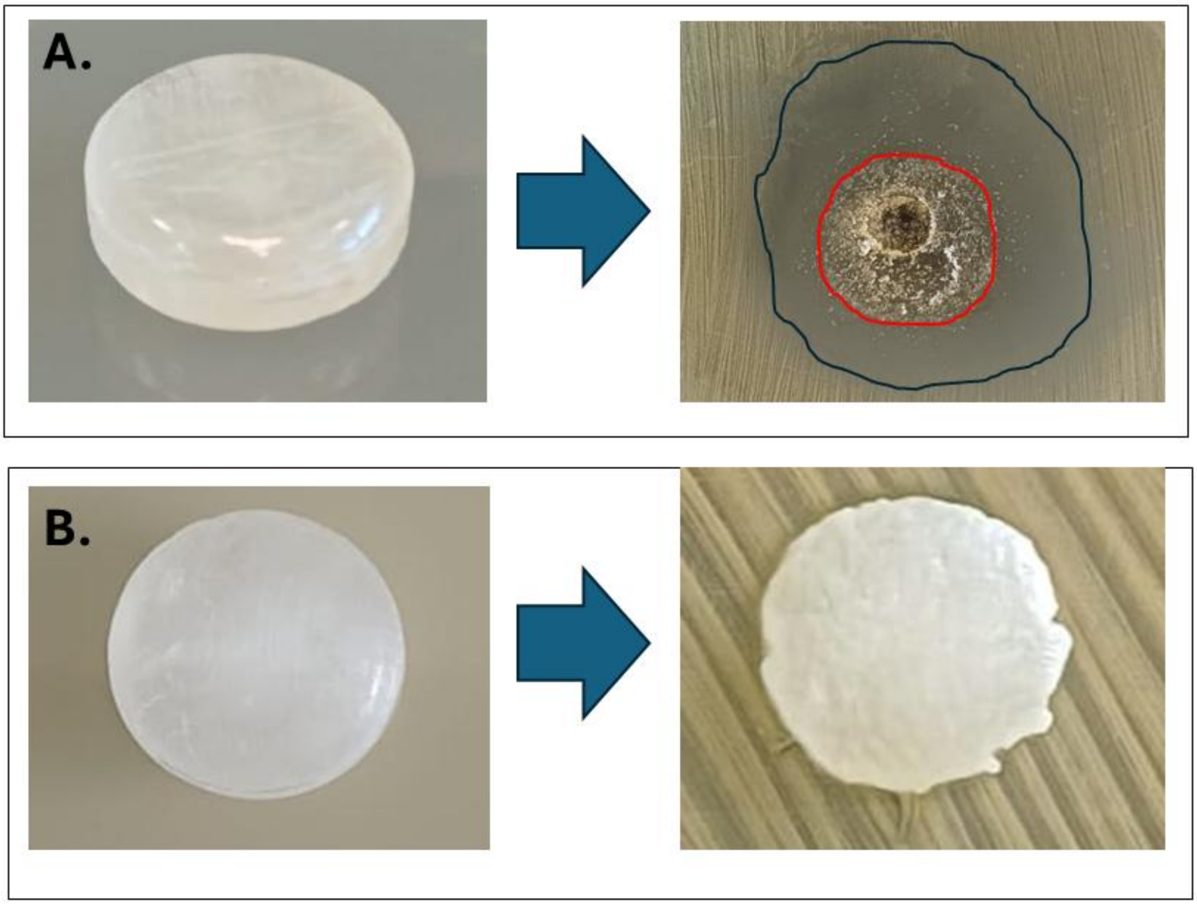

Figure S1. The set of control appied in MDDM method. **A**: lozenge containing another antiseptic agent (amylmetacresol) shows antistaphylococcal activity and serves as experiment's usability control. **B.** Lozenge containing all excepients but not octenidine – no stahylococcal growth inhibition is observed.
